# Convergent Transmission of RNAi Guide-Target Mismatch Information across Argonaute Internal Allosteric Network

**DOI:** 10.1371/journal.pcbi.1002693

**Published:** 2012-09-27

**Authors:** Thomas T. Joseph, Roman Osman

**Affiliations:** 1 Department of Structural and Chemical Biology, Mount Sinai School of Medicine, New York, New York, United States of America; 2 Computational Biology Program, New York University, New York, New York, United States of America; UNC Charlotte, United States of America

## Abstract

In RNA interference, a guide strand derived from a short dsRNA such as a microRNA (miRNA) is loaded into Argonaute, the central protein in the RNA Induced Silencing Complex (RISC) that silences messenger RNAs on a sequence-specific basis. The positions of any mismatched base pairs in an miRNA determine which Argonaute subtype is used. Subsequently, the Argonaute-guide complex binds and silences complementary target mRNAs; certain Argonautes cleave the target. Mismatches between guide strand and the target mRNA decrease cleavage efficiency. Thus, loading and silencing both require that signals about the presence of a mismatched base pair are communicated from the mismatch site to effector sites. These effector sites include the active site, to prevent target cleavage; the binding groove, to modify nucleic acid binding affinity; and surface allosteric sites, to control recruitment of additional proteins to form the RISC. To examine how such signals may be propagated, we analyzed the network of internal allosteric pathways in Argonaute exhibited through correlations of residue-residue interactions. The emerging network can be described as a set of pathways emanating from the core of the protein near the active site, distributed into the bulk of the protein, and converging upon a distributed cluster of surface residues. Nucleotides in the guide strand “seed region” have a stronger relationship with the protein than other nucleotides, concordant with their importance in sequence selectivity. Finally, any of several seed region guide-target mismatches cause certain Argonaute residues to have modified correlations with the rest of the protein. This arises from the aggregation of relatively small interaction correlation changes distributed across a large subset of residues. These residues are in effector sites: the active site, binding groove, and surface, implying that direct functional consequences of guide-target mismatches are mediated through the cumulative effects of a large number of internal allosteric pathways.

## Author Summary

Cells use the process of RNA interference (RNAi) to help orchestrate the production of the specific set of proteins needed at a given time. A given messenger RNA (mRNA) is produced using a gene in the cell's genomic DNA as a template. Each mRNA molecule is a blueprint for a particular protein and can then be “translated” into this protein. Prevention of translation is called “silencing”. An mRNA is chosen for silencing based on its sequence, through binding to a complementary “guide strand” which is itself bound to an Argonaute protein. Some Argonaute proteins have the ability to cleave the mRNA, silencing it. When the guide strand is not perfectly complementary to the target mRNA, silencing can still occur, albeit usually at a greatly reduced rate. Through molecular dynamics simulation and related methods, we examine potential explanations for this behavior with respect to the movements of the atoms comprising Argonaute. We show that there is a novel structural pathway through which a mismatch between guide strand and target mRNA can selectively affect certain functionally important parts of the Argonaute molecule, furthering our understanding of RNAi.

## Introduction

RNA interference (RNAi) is a fundamental mechanism for regulating the expression of genes in a variety of contexts. It is a process by which a short dsRNA, such as a short interfering RNA (siRNA) or microRNA (miRNA), can induce sequence-specific silencing of genes at the mRNA stage, preventing their translation into proteins. The short dsRNA contributes one of its strands, the guide strand, to bind with an Argonaute protein [1]. The resulting complex forms the central component in the multimeric RNA Induced Silencing Complex (RISC), which hybridizes to complementary mRNAs and silences them [2]. Although this is a sequence-specific process, guide and target need not be fully complementary, expanding the set of sequences that may be targeted by a single guide sequence [3]. The identity and position of any mismatched base pair variably affects the specific set of target genes and the extent to which they are silenced. This allows a single guide strand to potentially inactivate multiple proteins involved in multiple pathways, yielding wide-ranging effects. Discrimination of targets is accomplished partly through decreased binding affinity of the mismatched target; however, the inhibition of catalysis from the bound state may also play a significant role [4].

Mismatched base pairs also influence the assembly and maturation of RISC, which involves the loading of a short dsRNA into an Argonaute protein. Different Argonaute subtypes require the loaded dsRNA to have mismatches in different sequence positions for the recruitment of accessory proteins to form the finished RISC [5]. Correctly modifying the relative affinities of certain components of RISC for successful assembly hence depends on the ability of these accessory proteins to sense the presence of these mismatches.

Both of these functional aspects of Argonaute involve the remote sensing of a structural perturbation. In particular, a mismatched base pair must somehow inform other regions of the structure of its presence, producing the endpoint effect of modification of silencing activity. Information transfer of this nature is commonplace in biomacromolecules. It may take the form of a redistribution of the inherent conformational-energetic equilibrium of the structure: enhanced deformability of nucleic acids has been suggested to be a dynamical signal for sequence recognition by proteins [6], [7], mutations in aspartate transcarbamoylase produce distinct states in the conformational equilibrium [8], [9], and ligand binding to hen egg white lysozyme (HEWL) biases the conformational distribution as sampled computationally, stabilizing a particular helix, suggesting long range communication between the ligand and the helix [10].

In this work, we examine this intramolecular signal transduction by studying the Argonaute *ternary complex*, composed of an Argonaute protein bound to a guide-target nucleic acid hybrid. We examine the interaction correlation network as derived from molecular dynamics simulations of the *Thermus thermophilus* Argonaute ternary complex and determine the effect of introducing a single guide-target mismatch in each of several positions. Our methods relate residue dynamical couplings to residue energetic couplings, viewing the influence of the perturbing factor under question as a pathway that emanates from the site of perturbation and propagates information among different regions of the same structure [11]. Pathway approaches in general have the advantage that local correlations in atomic positions are easily observable on relatively short timescales such as those accessible through molecular dynamics simulation. (Relationships among residues may also be derived from nonphysical data: one method, operating on the assumption that coupled residue pairs are likely to be evolutionarily conserved, identifies groups of conserved residues and derives allosteric pathways from this information [12], [13].)

Using molecular dynamics simulation, we can study the atomic-level energetic fluctuations of the structure itself. We employ equal-time correlations of interaction energy fluctuations — the correlation between two sets of nonbonded interaction energies between pairs of amino acid residues — in order to capture the energetic basis of internal allosteric information transmission in Argonaute [14], [15]. Interaction energy correlations among residue pairs are mapped onto the constituent residues, and groups of residues defined by high correlation values with each other are regarded to comprise allosteric pathways. Previously, pathways have been identified using various operations on the residue correlation matrix, such as simple clustering [15] and graph theoretic methods like clique identification and shortest paths analysis [16]. Rhodopsin's conformational change upon photoactivation [17] was shown to be mediated by such an internal signaling pathway [14]. Ligand binding to PDZ2 causes a dynamical change that results in modification of its residue energetic couplings [15], which has been correlated experimentally by demonstration of changes in binding partner selectivity related to single residue mutations [18].

Our work builds on these previous efforts, which were successful for smaller proteins. However, in tightly packed proteins, as the number of residues *N* increases, the number of significant residue-residue interactions is proportional to *N* ^2^. This causes the number of significant correlations to increase substantially, making it difficult to identify distinct coherent narrow internal signaling pathways in large, tightly packed proteins such as Argonaute. In the present work, rather than identifying specific pathways, we instead determine the overall directionality of the relevant pathways as well as identifying specific endpoints. In this way, we provide evidence for a dynamical link between mismatched base pairs and Argonaute residues on the surface and near or in the active site. Even though the multimeric composition and organization of RISC is not presently known, a study of how Argonaute — as the central component of RISC from which an allosteric signal must originate — responds to mismatches in its nucleic-acid-containing complex can provide insight into these allosteric effects. We propose a model of internal allostery in which transmission of information is mediated by a distributed set of intermediate residues, such that a small number of key residues are perturbed by any of several different small structural perturbations. We undertake a study that does not have a convenient experimental equivalent, in order to better inform present and future experimental models.

## Results

We used the interaction correlation method [14], described in the Methods section, to determine the pattern of energetically coupled pathways in *Thermus thermophilus* Argonaute (TtAgo), with and without a bound nucleic acid substrate. (We refer to Argonaute bound to a single guide nucleic acid strand as the *binary complex* and to Argonaute bound to a guide-target nucleic acid hybrid as the *ternary complex*.)

This method computes the pairwise residue interaction energies of many conformational states of the structures at thermal equilibrium sampled from a MD trajectory. The interaction energy calculations include only nonbonded terms from the force field equation: van der Waals (*i.e.* Lennard-Jones terms) and electrostatic interaction energies, which are added together to produce the total interaction energy. Because electrostatic interactions decay over a much longer distance compared to van der Waals interactions, electrostatic energies dominate the calculated quantities. As an example, for a representative ternary complex snapshot, the magnitude of the sum of all pairwise residue electrostatic interaction energies was on the order of 30,000 kcal/mol. By comparison, the sum of the corresponding van der Waals energies was approximately 3400 kcal/mol. It is possible that at relatively short ranges the two terms may be of similar magnitude and opposite signs and as such cancel each other out, masking significant interaction changes. In the case of the fully-matched TtAgo ternary complex, in a given trajectory snapshot, there were in general less than 50 residue pairs in which this situation occurred, as defined by energy magnitudes greater than 0.5 kcal/mol (in most cases less than 2 kcal/mol) and sum less than 0.1 kcal/mol. See Supporting Table S1 for example energy values from a representative snapshot. For the purposes of this study, we felt that the individual contributions of van der Waals and electrostatic energies were not essential to the conclusions, and so we chose to examine the nonbonded energies as determined using the force field equation.

**Figure 1:**
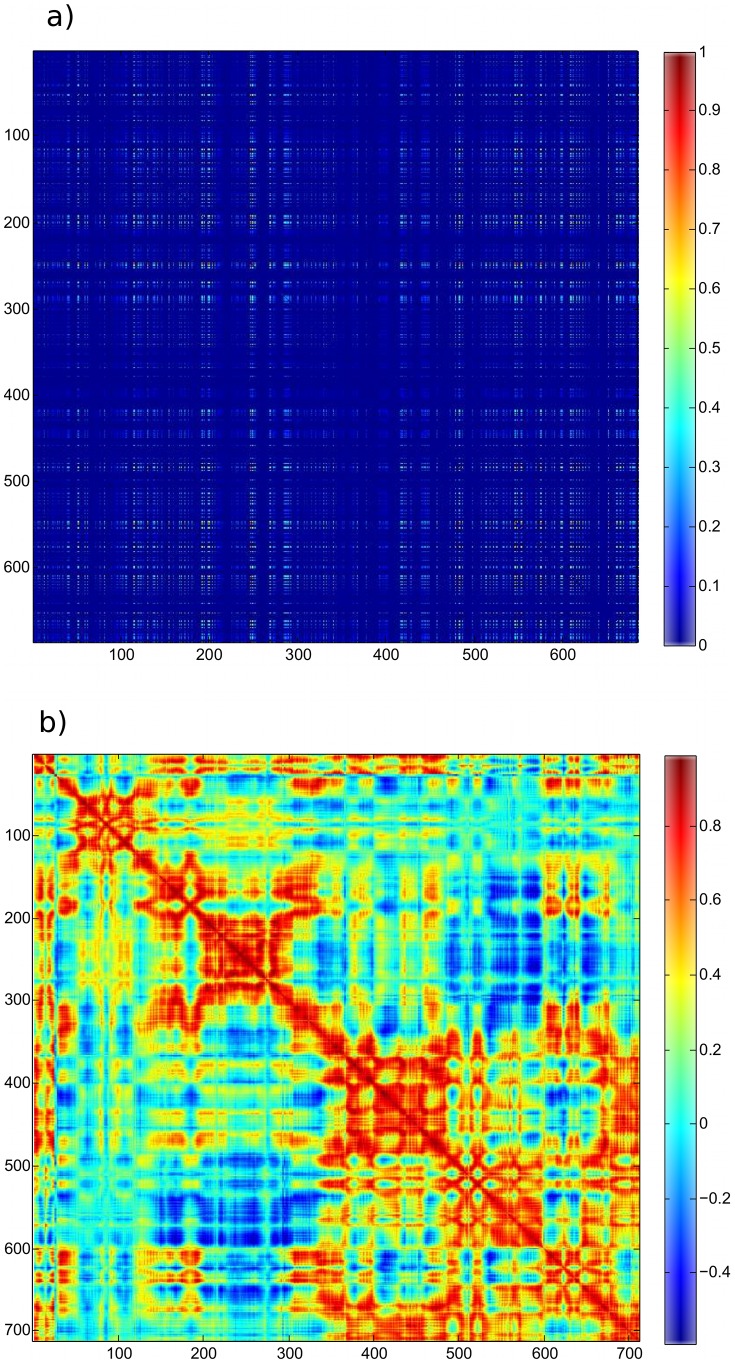
Residue correlation versus positional correlation matrices. (a) Residue correlation matrix for the TtAgo 3F73 fully-matched ternary structure, with bound nucleic acid omitted. Blue represents zero (uncorrelated) and red represents one (fully correlated). Note the heterogeneous distribution of high residue correlation values spanning the structure, indicating a large number of difficult-to-discern internal allosteric pathways. (b) Positional correlation matrix for the same structure provided for comparison.

Correlations among pairs of residue interaction energies were calculated and subsequently projected back on the structure to create a residue correlation matrix where each axis represents the protein sequence (see Figure 1). Each matrix cell then describes the degree of energetic coupling between two residues. A row or a column in the (symmetric, by construction) residue correlation matrix corresponds to a vector of the correlations for a specific residue with all the other residues. Importantly, the residue correlation matrix, by describing the correlations among *pairs* of interactions, goes beyond the calculation of direct interaction or positional correlations among *single* residues. This analysis is distinct from position correlation analysis; the position correlation matrix for the same trajectory is shown in Figure 1 for comparison. The residue correlation matrix may change when there is a structural modification; this is illustrated in the binding of nucleic acid to the free TtAgo to form a ternary complex, which results in a relatively small but orderly change in the residue correlation matrix. The delta between the respective residue correlation matrices for this example is shown in Supporting Figure S1.

Notably, the qualitative features of residue correlation matrices discussed below manifested similarly at both relatively short (6–12 ns) and long (80–100 ns) timescales, suggesting good convergence at short timescales. It is important to note that correlations at timescales considerably longer than we were able to obtain are likely to be important, possibly resulting in the determination of an internal allosteric network modified from the one we present here; for example, partially connected networks or a fundamentally different architecture could emerge. Of course, the possibility of inadequacy due to sampling limitations is inherent to any study depending on MD simulation. Regardless, it is a strength of this method that we can still glean much important information from short-timescale correlations easily accessible through MD.

### Interaction correlation network is centered on active site

Examining the residue correlation matrix for the TtAgo ternary complex shows that there is a widely distributed set of correlations among residues in all regions of the protein. This is shown in Figure 1a: note the widely distributed regions of high correlation values. This represents an organized set of interaction pathways, described by energetic couplings, that spans the structure. Residues that are part of the same pathway would have high correlation values with each other. However, specific pathways do not present themselves as an obvious property of the matrix.

Previous workers had identified pathways by regarding the residue correlation matrix as a similarity matrix and using clustering methods to identify groups of residues associated by large correlation values with each other. We repeated this approach, using the Markov Cluster algorithm (MCL) [19], as previously used with a smaller protein [15], and subsequently, average-linkage divisive hierarchical clustering [20]. However, with both methods, as the clustering granularity was progressively decreased, large numbers of singleton clusters were resolved. These methods did not identify a pathway (or pathways) but rather suggested that in the setting of a residue correlation matrix densely populated with high similarity values, there are many interaction correlation pathways that are not clearly distinct from one another. This is consistent with the fact that Argonaute is a large protein with a large number of residue-residue interactions, and highlights the difficulty of isolating individual internal allosteric pathways in proteins of this large size.

**Figure 2:**
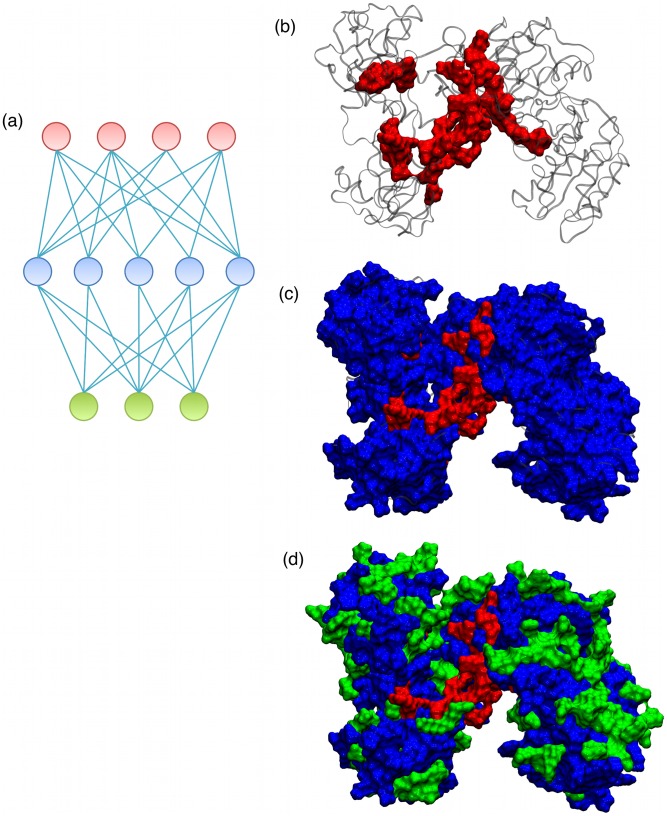
Illustrations of residue correlation matrix clustering. The diagram in (a) shows three clusters. Each circle represents a single residue. The color of a given circle denotes cluster assignment. Light blue lines indicate strong residue correlation (i.e. a large *M_ij_* value; see Methods). Note that a cluster is defined by its pattern of correlations. The three illustrations (b) through (d) show each of these three cluster groupings in the TtAgo ternary complex. Note red cluster comprises the core; blue comprises the bulk, and green includes some surface residues.

We next took a different approach to identifying pathway structure, employing a different similarity metric while retaining the use of average-linkage divisive hierarchical clustering. In this approach, the residue correlation matrix is treated as a matrix of observations by residue, where each “observation” for a given residue represents the degree of correlation with another particular residue. Starting with one large cluster containing all residues, each cluster is then recursively divided by choosing the cluster with the greatest internal dissimilarity at each step. In this way, a given pair of residues would only be assigned to the same cluster if they both correlated strongly to similar subsets of residues. Importantly, this pair of residues need not actually have a strong correlation with each other. With this metric, the meaning of a given cluster is that it is a “slice” across a set of correlation pathways rather than a discrete pathway. This is illustrated schematically in Figure 2a, where each circle represents a residue and each connecting line represents a high correlation value between two residues. Note that the red residues at the bottom of the diagram are strongly connected to the blue residues, but are not connected to each other. Hence they are grouped together. Similarly, the blue residues connect strongly to the red residues and the green residues, and as such are grouped together. Finally, the green residues connect primarily to the blue residues and are grouped together. Note that clustering in this manner does not resolve individual correlation pathways, yet the overall directionality of the network is evident. Although the specific clusters chosen are to some degree dependent on the clustering method and as such the set of correlations evident in the partitioning is not necessarily exhaustive, this clustering provides substantial insight into the overall organization governing transfer of information within the interaction correlation network.

Applying this concept to partitioning the residue correlation matrix for each structure, we produced a set of cross-sections of the interaction correlation network in the Argonaute ternary complex, which revealed the overall architecture of the network. In all matched and mismatched ternary complexes, we found that there were roughly six major clusters of residues that were robustly prominent at a variety of clustering levels. These clusters were organized in layers emanating from the active site region (*i.e.* corresponding to the red cluster in Figure 2a), in an “onion-skin” type architecture (see Figure 2b–d). The largest cluster included the cores of all protein domains (blue cluster), showing that they are the common link in all emanating pathways. This cluster was surrounded by a distributed cluster (green cluster) consisting of residues on the protein surface, with which it directly interacts. The central clusters (red) include the active site and the target scissile bond. This architecture allows dynamical signals to propagate from the active site region (red), through the protein domain cores (blue), onward to the surface of the complex (green), and *vice versa*. This widely-distributed but non-uniform network of interaction pathways follows from the architecture of Argonaute because all domains, although structurally well defined, are in relatively close proximity to each other and therefore their nonbonded interaction energies tend to be well correlated. Such architecture allows the dynamics of any given region of the complex to influence all other regions of the complex. This would allow Argonaute to support allosteric interactions that would have distant endpoint effects at many regions across its surface.

### Influence of mismatch depends on its sequence position

We also examined certain individual elements of the interaction correlation network. Since the guide strand seed region has been implicated in RNAi sequence selectivity, we hypothesized that in a ternary complex, the degree to which a given guide strand nucleotide can influence Argonaute protein residues would depend on its position. The rationale for such a hypothesis is based on a structural argument related to the nature of nucleic acid protein interactions. In the ternary complex, as one progresses along the nucleic acid sequence (*e.g.*, from position 3 towards position 11 near the active site) the base pairs are not only translated from the anchoring site towards the active site, but they also change their orientation with respect to the protein because of the helical nature of the nucleic acid. In approximately 5 base pair steps, the orientation turns ∼180°, such that if the guide strand was proximal to the protein, after 5 base pairs, the target strand is now in an equivalent, albeit translated, position. To some extent this was the origin of sequence-position-dependent changes in the free energy of introducing a mismatch, as was observed in our previous work [21].

**Figure 3:**
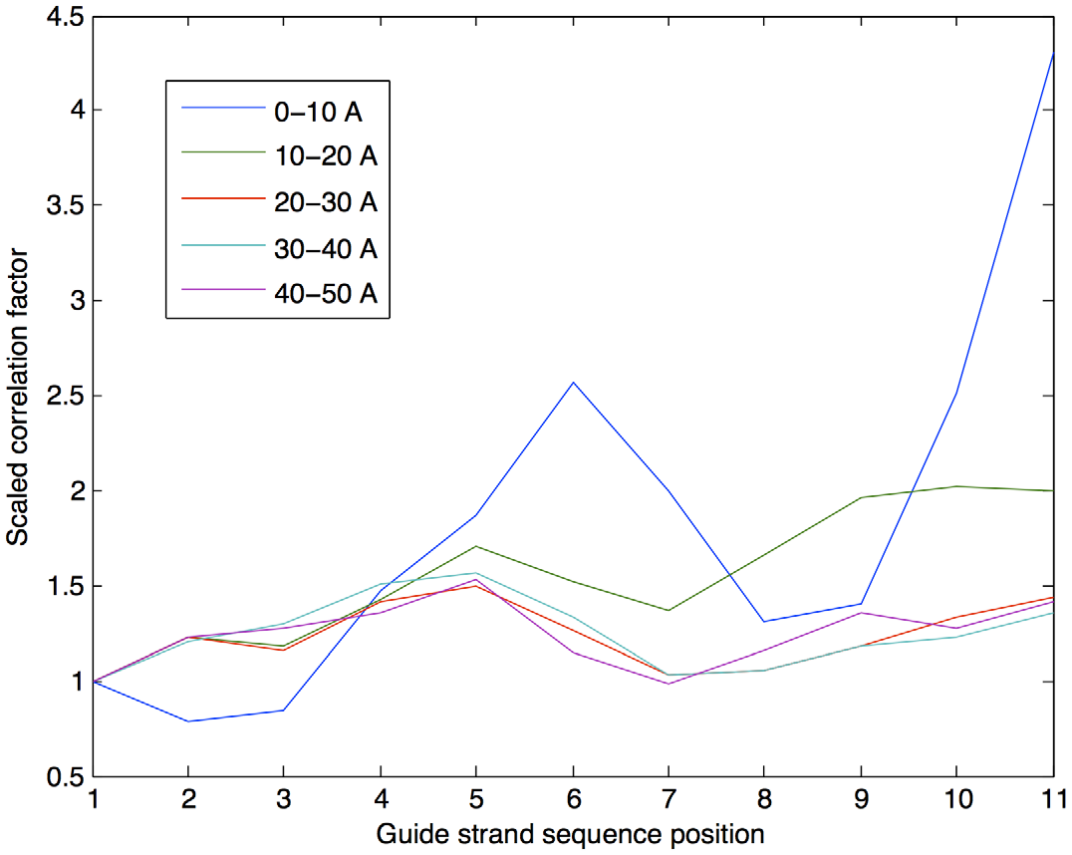
Relative correlation factor by distance in fully-matched TtAgo ternary complex. For clarity, the correlation factor for each distance shell is normalized by its value in guide strand position 1. Note that cleavage site residues have strong effect on nearby protein residues, but as distance from the nucleotide is increased, this effect is decreased, and the effect of nucleotides 3–6 becomes relatively more prominent. This suggests a long-range effect on internal allosteric pathways of the seed region, but only a local effect of the active site. Similar results are obtained for all mismatched ternary complexes. Distance is in Ångstroms.

To probe this coupling, we calculated the *correlation factor* of each nucleotide with the protein as detailed in the Methods section. Essentially, this is the sum total of correlation values of the nucleotide with the rest of the protein, providing a description of the degree to which interaction pathways connect each individual nucleotide with the protein. In all TtAgo ternary complexes, guide strand positions 4–6 (part of the seed region) have increased correlation with the protein regardless of the presence of a mismatch. The increased seed region correlation suggests that while structural distortion is minimal when there is a guide-target mismatch in the seed region, it has the potential to propagate a signal to the protein to a greater degree than a mismatch in the surrounding positions. We also probed the dependence of the correlation factor on distance from the nucleotide (see Figure 3). Interestingly, active site nucleotides (the scissile bond is between nucleotides 10 and 11) had a large correlation factor with respect to nearby protein residues, but this is greatly diminished with distance, indicating that perturbations in these residues have primarily local effects. By contrast, seed region nucleotides had a prominent effect in distant protein residues (30–50 Å). This is congruent with existing theories stating that the seed region is the primary determinant of target selectivity, because a seed region mismatch is better able to influence the rest of the structure than a non-seed-region mismatch. This is also congruent with the hypothesis that this binding free energy penalty is the primary mechanism through which mismatches in the seed region reduce RNAi cleavage activity, consistent with the results of experiments showing a decrease in RNAi catalytic efficiency due to seed region mismatches [4], [22].

### Whole-network effects of guide-target mismatches in the nucleic acid

Previously, we found in simulations that a guide-target mismatch tends to destabilize the ternary complex relative to the binary complex, which would disfavor target binding [21]. Examining the simulation trajectories of all mismatched TtAgo ternary complexes showed that in each case, significant structural distortion was limited to the mismatched base pairs themselves and their close neighborhood. In addition, for all simulations of the mismatched ternary complexes, we observed that the RMSD was less than 3 Å with respect to the fully-matched ternary complex. This suggests that the dynamical perturbations emanating from the mismatch site do not cause a large structural change even though their energetic change is quite substantial and highly significant with respect to function. We wished to determine how this energetic change affected the interaction correlation network. To do so, we compared the residue correlation matrix of the fully-matched TtAgo ternary complex with those of mismatched TtAgo ternary complexes. To determine whether gross interaction pathway reorganization took place within the protein, we designed and used a similarity metric designed to describe the degree to which the general pattern of internal correlation pathways is preserved when a mismatch is introduced. The method is described in detail in the Methods section, and is briefly described below.

We were primarily interested in the degree to which strong correlations were preserved or lost across the two structures in a given comparison, so we excluded from consideration all but the strong correlations. In order to identify these strong correlations for a given residue correlation matrix, we plotted all correlation values, extracted from the matrix, in ascending order. This resulted in a curve with two distinct regions — a low-valued region representing small correlations and a high-valued region representing strong correlations (see Supporting Figure S2). In all cases, the high-valued region represented the top 7.4% of values, which for a given residue correlation matrix was approximately 17350 distinct correlation values, out of (*N* ^2^−*N*)/2 = 234270 possible (given that the residue correlation matrix is symmetric). To compare two residue correlation matrices *A* and *B*, each cell at *A*_ij_ was examined along with its corresponding cell *B*_ij_ to produce a combined similarity score (see Methods). Each corresponding pair where both had strong correlations in the top 7.4% subset contributed a 1 and those correlations that were unmatched contributed a zero. Those correlations that were not in the top 7.4% subset were excluded. The similarity score was therefore the fraction of the corresponding pairs out of the total number of correlations preserved in the top 7.4%. The intuition underlying this method is that in the case of full preservation of the interaction correlation network, if there is a strong correlation between residues *i* and *j* in a fully-matched structure, then *i* and *j* should also have a strong correlation in the mismatched structure, and vice versa. Each score was compared to an expected similarity score between randomized versions of the same two matrices in order to assess for significance (see Methods). In all cases only the Argonaute protein was included in the comparison.

Tables 1 and 2 show comparisons using the similarity score between various matched and mismatched ternary complexes. Every 0.001 increment in the similarity score represents approximately 35 modified strong interactions. The maximum possible similarity score is 1, indicating a fully-preserved allosteric network architecture that would preserve the dynamics of surface residues interacting with allosteric partners. The similarity scores between matched and mismatched ternary complexes are in the range 0.9904–0.9911, which implies that there were on the order of 300 modified strong correlations in each case. Comparing the mismatched ternary complex residue correlation matrices to each other resulted in similarity scores ranging from 0.9977 to 0.9987 (see Table 2). This indicates that the different mismatched ternary complexes are more similar to each other than they are to the fully-matched ternary complex. For all comparisons the similarity scores were substantially higher than the randomized value of approximately 0.1514, indicating that they were significant.

**Table 1:** Residue correlation matrix similarity scores — protein subsets only.

TtAgo (3HK2)+fully-matched dsNA	vs.	TtAgo (3HK2) protein only	0.9964
		TtAgo (3HK2)+guide strand only	0.9970
		TtAgo (3F73)+truncated fully-matched dsNA	0.9908
TtAgo (3F73)+truncated fully-matched dsNA	vs.	TtAgo (3F73) protein only	0.9913

Only the top 7.4% of correlation values were considered. The expected score distribution for all comparisons had mean 0.1514 and standard deviation less than 0.001.

**Table 2:** Residue correlation matrix similarity scores — ternary complexes, protein subsets only.

	Fully-matched	G3T	A4C	A4T	G6C	T7G
**Fully-matched**	1					
G3T	0.9911	1				
A4C	0.9909	0.9986	1			
A4T	0.9906	0.9982	0.9984	1		
G6C	0.9906	0.9982	0.9986	0.9977	1	
T7G	0.9912	0.9984	0.9986	0.9979	0.9987	1

All complexes derived from structure with PDB ID 3F73.

These high similarity scores suggest that the architecture of the network is quite resilient to perturbations, whether they originate from a binding event, a conformational change or the introduction of a mismatch in the nucleic acid sequence. We were most interested in the ∼1% of variability in the network arising from a guide-target mismatch. Since the only structural difference between matched and mismatched ternary complexes was within the mismatched base pair itself, this dissimilarity can be considered to arise from the presence of the mismatched base pair. We therefore designed an approach to identify specific residues affected by these structural perturbations.

### Argonaute residues preferentially affected by guide-target mismatches

We observed that a single mismatch can modify the entire interaction correlation network, causing a subset of residues to be either coupled or decoupled from the network. Given the pattern of similarity scores, we hypothesized that this subset would have significant commonality across mismatched ternary structures. To identify these common elements, we examined each *difference matrix* resulting from element-wise subtraction of the fully-matched residue correlation matrix from each mismatched residue correlation matrix. To facilitate comparisons among different matrices, each residue correlation matrix was first converted to units of standard deviations from its element mean, and only the Argonaute protein was included in these calculations. For each residue correlation matrix *M*_mismatched_ corresponding to a given mismatched ternary structure, we calculated Δ*M* = *M*_mismatched_−*M*_matched_. Therefore, each cell in the difference matrix Δ*M_ij_* describes the change in correlation value between residues *i* and *j* due to the presence of a mismatch. For a given residue pair, a gain in correlation would suggest that new interaction pathways connected to the perturbation pass through this coupling, and a loss would suggest that such pathways were removed. In both cases, this means that the dynamics of a residue pair are modified in response to the perturbation. However, we were interested in the average magnitudes of differences rather than their signs, so we calculated the root mean square of each cell Δ*M*_ij_ across all difference matrices. This resulted in an averaged difference matrix Δ*M*_rms_.

**Figure 4:**
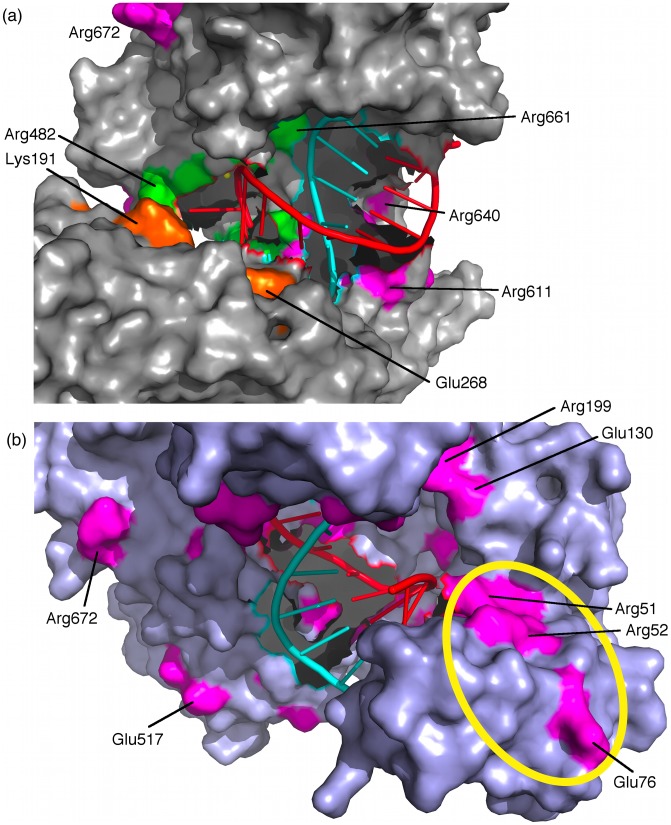
Sensor residues of TtAgo. Selected sensor residues are labeled. (a) Binding groove sensor residues. Guide DNA strand in cyan, target RNA strand in red, sensor residues proximal to active site in orange, PIWI binding groove sensor residues in green, other sensor residues in magenta. Note the preponderance of sensor residues in the nucleic acid binding groove. (b) Putative target RNA interaction region, encircled in yellow, shown in 3HK2 TtAgo ternary complex.

To quantitate the degree of involvement of each residue in the modification of the residue correlation matrix due to the introduction of any of the tested mismatches, we summed the values in each column of Δ*M*_rms_ to produce a vector where the *i*th element describes the amount of change in correlation value for residue *i* combined across other residues in the structure. The largest values in this vector corresponded to residues which had their coupling to the interaction correlation network most affected by the mismatches. Importantly, a large value could result from both increases and decreases in correlation value. To determine the most important residues by this measure, we identified the 65 elements of this vector that were more than two standard deviations away from the mean of all 685 elements in the vector, and the corresponding residues were deemed the *sensor residues*. These sensor residues gain or lose coupling with the rest of the structure in the presence of a seed region guide-target mismatch. The sensor residues are listed in Table 3 and diagrammed in Figure 4.

**Table 3:** Sensor residues in TtAgo ternary complex.

Index	Type	SASA	Salt bridge
51	Arg	127	
52	Arg	118	
76	Glu	111	Arg89
114	Arg	93	Asp154
115	Arg	18	
130	Glu	23	Arg172*, Arg199*
166	Glu	50	
172	Arg	37	Glu130
0191	Lys	135	Glu203
192	Arg	94	Glu203
194	Arg	26	
199	Arg	110	Glu130
200	Arg	181	Asp198
203	Glu	10	Lys191*, Arg192*
246	Asp	81	
248	Lys	157	
249	Asp	75	Arg251
268	Glu	79	
269	Asp	64	
289	Arg	95	Glu285
335	Arg	96	Glu448
340	Arg	142	
478	Asn	0	
482	Arg	152	
517	Glu	92	
521	Asp	39	Arg513
546	Asp	19	
548	Arg	44	
552	Asp	133	
553	Glu	37	
574	Arg	119	
575	Lys	84	
597	Glu	94	Arg13
598	Asp	147	
608	Arg	64	
609	Asp	96	
611	Arg	42	
618	Lys	49	
640	Arg	23	Asp590
651	Arg	9	
660	Asp	15	
661	Arg	29	
665	Glu	8	Arg418, Arg668
672	Arg	204	
685	Val	13	

Solvent-accessible surface area, reported in Å^2^, was calculated with a probe radius of 1.4 Å. Certain sensor residues are taken to form salt bridges by distance (O–N distance cutoff 3.2 Å in the 3F73 crystal structure); asterisks denote salt bridges between sensor residues.

Sensor residues were found in N-terminal, PAZ, and PIWI domains as well as the linker regions, but none were found in the Mid domain. Since the Mid-PIWI interface binds the 5′ end of the guide strand, a short distance from the seed region, this shows that the distribution of sensor residues was nonuniform. Nearly all sensor residues were charged, with a predominance of arginine and glutamic acid residues, as well as several aspartic acid and lysine residues. Since the interaction energies are dominated by electrostatics, the fact that so many sensor residues were charged is therefore not surprising. Some sensor residues formed salt bridges, which could in principle change orientation, serving as an allosteric mechanism; these are listed in Table 3. The sensor residues could be divided into three categories: two residues forming part of the catalytic triad, binding groove residues, and surface residues.

The active site residues D546 and D660, which interact with both Mg^2+^ ions in the active site, were found to be sensor residues. This suggests an effect of seed region mismatches directly upon the target cleavage reaction. It is likely that the mechanism of target cleavage in Argonaute is similar to that of RNase H given the structural similarities between the respective active sites — indeed, we placed the Mg^2+^ ions in the system by analogy with RNase H (see Methods). In the RNase H mechanism, an Mg^2+^ ion is likely to catalyze the nucleophilic attack of the scissile phosphate by a water, forming an intermediate which is bound to the other Mg^2+^ ion. Protonation of the leaving group by an active site Asp residue completes the reaction. During the course of the reaction, the inter-Mg^2+^ distance is modified, suggesting that the motion of these ions is significant [23]. Disrupting any of these components, whether an Asp itself or an Mg^2+^ complexed to the Asp residues, could potentially modify the catalytic rate. In addition, the binding groove sensor residues, described below, may be part of internal allosteric pathways terminating at D546 and D660. The identification of these residues strongly suggests a focused effect on the active site region due to seed region guide-target mismatches.

Interestingly, much of the length of the Argonaute binding groove was composed of sensor residues, demonstrating a long-range effect of seed region mismatches upon the binding groove. This included a subset of residues that were located proximal to the active site; these were K191, R192, R194, E203, D246, K248, D249, and E268; this region is illustrated in Figure 4a. Several binding groove sensor residues were part of the PIWI domain: R482, R574, K575, K618, D546, R548, E597, D598, D660, R661, E665, and R685, also illustrated in Figure 4a. Even though we employed a TtAgo guide-target structure in which both strands of the bound nucleic acid were truncated after guide strand position 12, there were a number of sensor residues in the regions of the binding groove that would normally bind the missing nucleotides. This suggests that the entire binding groove — both guide- and target-binding regions — is dynamically coupled to the seed region, and that a seed region guide-target mismatch may have an effect upon the interface between protein and nucleic acid, even in regions relatively distant from the site of the mismatch.

In order to estimate more accurately how the binding groove sensor residues were positioned relative to the “missing” nucleotides, we identified the sensor residues on a TtAgo ternary structure in which the missing nucleotides were resolved, but for which no experimental data regarding target cleavage as a function of mismatch is available [24] (PDB: 3HK2). Binding groove sensor residues were found to be in close relationship to the “missing” nucleotides (see Figure 4b). Notably, near the 5′ end of the target RNA, there is a strip of sensor residues which follow the putative path of a longer target RNA as it would extend into the TtAgo protein. This lends credence to the idea that the Argonaute-target interface is modified along its entire length by an internal allosteric mechanism originating at seed region guide-target mismatches.

Although the location of binding of RISC accessory proteins to Argonaute is not known, sensor residues on the surface of Argonaute may mediate the passage of the internal allosteric signal arising from the mismatched base pair to the surface of the protein, and by virtue of their physical accessibility to external molecules would represent potential external allosteric interfaces. We wished to identify these sensor residues because they may mediate the recognition of mismatches by external proteins, such as Dicer or accessory proteins in RISC. We defined a surface sensor residue as having relatively large solvent-accessible surface area (>70 Å^2^), as calculated using a standard probe radius of 1.4 Å, without being located in the binding groove. (The solvent-accessible surface area values are given in Table 3 and diagrammed in Supporting Figure S3.) These residues were E76, R289, R335, R340, E517, and D521. There were relatively few regions containing such surface sensor residues. Surface sensor residues R335 and R340 are located in the PIWI domain, on the opposite side of the protein from the binding groove. E517 and D521 are located adjacent to each other in the PIWI domain. R289 is located near the 3′ end of guide strand. E76 is in the N-terminal domain.

In general, the locations of the sensor residues are congruent with our description of the “onion skin” architecture of the internal allosteric network with the active site at the center: the sensor residues near the active site are part of the central clusters, and those on the surface are part of peripheral clusters. The overall picture is of three general categories of sensor residues with apparent functional importance: residues on the surface of the protein, which can transmit a signal to external binding partners; residues in the binding groove, which can modify the interface along the length of the bound nucleic acid distant from the seed region mismatch; and residues near and in the active site, which could potentially affect target cleavage.

## Discussion

We present here evidence that internal allosteric signaling pathways propagate diffusely in Argonaute but can converge to produce focused distal allosteric effects, mediated through the aggregation of many small changes. Methodologically, we show that key residues in internal signaling pathways can be identified by their dynamical response to a structural perturbation such as a guide-target mismatch. Functionally, we show that in TtAgo these residues are located in mechanistically important regions.

Prior applications of the interaction correlation method have generally been with comparatively small structures such as PDZ2 [15] and pyrrolsyl-tRNA synthetase [16], in which discrete internal allosteric pathways were identified, progressing from origin to endpoint along a narrow pathway. Our analysis of Argonaute, a considerably larger protein, is consistent with a description of internal allosterics that is different but complementary — that a coherent and consistent signal acting on a small set of residues is propagated through a wide cross-section of residues which converges on these residues rather than through a small set of narrow pathways. We found that the general architecture of the internal allosteric network is preserved in structures with guide-target mismatches, but that a number of key residues were consistently affected even by different mismatches. This demonstrates a convergent common pathway with specific functionally relevant endpoints in Argonaute, which is a novel finding.

In particular, the identification of catalytic residues D546 and D660 as sensor residues suggests the existence of an internal allosteric link between seed region mismatches and the mechanism of target cleavage. This result is consistent with experimental evidence; it is well known that guide-target mismatches can impair silencing activity [4], [24]. Although seed region mismatches were shown experimentally to primarily affect the binding of the target strand (*i.e.* by modifying the Michaelis-Menten parameter *K*_m_), there was still some effect on the rate constant [4], compatible with the hypothesis that when the mismatched ternary complex forms, target cleavage is impaired. This pathway is a potential explanation for this behavior, and the first suggestion of an internal allosteric link between seed region mismatches and the active site region.

The effect upon the binding groove was pronounced. Importantly, the contact regions for both the guide and target strands were affected. We show here that the dynamics of the interface between Argonaute and bound nucleic acid can be modified by a seed region guide-target mismatch. We previously showed in simulations that guide-target mismatches could increase the affinity of the double-stranded nucleic acid to Argonaute while simultaneously favoring the dissociation of the target strand. Here, we have shown a direct dynamical correlate of this finding.

We can also speculate regarding a role of sensor residues in the context of internal allostery in RISC formation and maturation. During this process, Dicer transfers an miRNA to Argonaute [25] and then must dissociate to allow separation of the passenger strand. The precise mechanism for this process is unknown, but Dicer must be prevented from dissociating before the transfer is complete. We speculate that a dynamical signal originating from the presence of the newly loaded nucleic acid, propagated via the ordered binding site and further onward to the surface through intra-complex correlation pathways, could promote Dicer dissociation as loading completes. In *Drosophila*, miRNA-containing Ago1 and Ago2 pre-RISC complexes require mismatches in specific ranges of the miRNA in order to mature to their respective functional RISCs [5]. Hence there is likely to be an allosteric modulation step that either promotes or represses RISC maturation depending on the mismatch pattern of the bound miRNA. In the latter case, an “incorrect” pattern of internal mismatches can inhibit the conversion of a pre-RISC into a mature RISC [5]. A dynamical mechanism would require a signal to be passed from the mismatch sites along the pervasive interaction correlation pathways to allosteric partners.

The identification of specific residues in which dynamical changes are manifested suggests a method for experimental validation. Mutation of a functionally relevant sensor residue should to some degree disrupt the dynamical changes due to the perturbation (in this case, a mismatched base pair). If these dynamical changes are important to a mechanism, such as in allosteric interaction with an external partner, that mechanism would be disrupted as a result. Most of the sensor residues were of non-neutral charge, suggesting that strong electrostatic interactions are characteristic of sensor residues. We found that a structural perturbation that results in no net change in charge — a base substitution — can result in an electrostatic change, by way of perturbation of the dynamics of a charged residue. Hence, changing the sign of the charge of a charged sensor residue or substituting a neutral residue would be expected to have the greatest effect.

As discussed above, the vast majority of sensor residues identified were charged, reflecting the nature of our energy calculations. Extending our analysis to include solvation energies in addition to electrostatic and Lennard-Jones energies may increase the number and variety of sensor residues identified. Employing entirely different analytical strategies, whether MD-based or otherwise, may provide further insight, although we felt that such exhaustive work was outside the scope of this study. For example, residue coevolution methods [12], [13] may provide corroborating results, although the sequence disparity of Argonaute proteins for which structures are available (*e.g.* TtAgo against *Aquifex aeolicus* Argonaute has 12% sequence similarity using the Gonnet score matrix, calculated using ClustalW2 with default parameters [26], [27]) may make these methods difficult to use. Finally, analyzing other Argonaute structures would also be helpful in constructing a more general characterization of Argonaute internal allosteric networks. Nonetheless, our analysis presents a compelling view of the internal allosteric effects of the introduction of a guide-target mismatch into the Argonaute ternary complex, with substantial relevance to the structural and dynamical basis of the mechanism of RNAi.

## Methods

### Preparation of structures

The Argonaute-guide-target structure (*ternary complex*) from which the main structures simulated were derived was prepared from a crystal structure of *T. thermophilus* Argonaute (TtAgo) loaded with a guide-target DNA•RNA hybrid [28] (PDB accession number 3F73). Although other TtAgo crystal structures are available, this particular structure of a TtAgo complex was selected because of the availability of experimental data relating single seed-region guide-target mismatches in the ternary complex to decreased target cleavage rates. In order to approximate a catalytically competent configuration, two Mg^2+^ ions were placed in the active site by analogy with the active site configuration of an RNase H complex bound to a DNA•RNA hybrid [29] (PDB accession number 1ZBI). The Argonaute-guide structure (*binary complex*) was constructed by removing the target strand and Mg^2+^ ions from the ternary complex. Loops unresolved in the crystal structures, all distant from the catalytic region and binding groove, were predicted using MODELLER [30]. The nucleic-acid-only structure was constructed by removing the Argonaute and Mg^2+^ ions from the ternary complex. All structures were solvated with explicit waters in a truncated octahedral box and neutralized with Na^+^ ions. Mismatched structures were produced by manually mutating the base in question on the guide strand. Each mismatched base pair conformation was constructed by analogy with similar base pairs in the Non-canonical Base Pair Database [31], subject to the necessity of avoiding steric clashes with the surrounding structure.

The force fields employed were the AMBER99SB force field with ParmBSC0 nucleic acid corrections [32], TIP3P waters [33], and the MD6 dummy-atom Mg^2+^ ion representation [34]. ParmBSC0 was developed to alleviate nucleic acid structural distortions that appear at long timescales when AMBER99-family force fields are used. The MD6 model was developed to improve the accuracy of simulations of DNA polymerases by alleviating inaccurate active-site distortion caused by repulsive forces between two adjacent Mg^2+^ point charges. It was shown by its authors to accurately reproduce crystal structures for DNA polymerase β — an enzyme which, like RNase H and Argonaute, catalyzes a phosphoryl transfer reaction — while the point charge Mg^2+^ model introduced significant distortions. The use of MD6 also allowed calculations of free energy of binding of dNTPs to DNA polymerase β that were in stronger agreement with experimental results compared to the same calculation with the point charge Mg^2+^ model. Since ParmBSC0 and MD6 address force field shortcomings that would affect key structural elements in our simulations, we expected the use of these parameters to improve their accuracy.

### Simulation and analysis protocol

We conducted molecular dynamics simulations of crystal structures of TtAgo bound to a truncated DNA:RNA hybrid (dsNA) (3F73), TtAgo bound to several mismatched, truncated dsNAs (derived from 3F73); and for comparison free TtAgo (TtAgo from PDB accession number 3HK2 with nucleic acid removed) and TtAgo bound to a 21 base pair fully-matched dsNA (3HK2). The 3HK2 structure resolves more bound nucleotides distal to the seed region than the 3F73 structure (19 bp vs 12 bp).

A preliminary version of the AMBER 11 biomolecular simulation package [35] was used for simulations. A separate minimization, heating (NVT), and equilibration (NVT followed by NPT) protocol was conducted. The solute was position-restrained with harmonic force constant 5 kcal/mol during minimization and heating. This restraint was gradually released during NVT equilibration. Periodic boundary conditions with the particle mesh Ewald method for evaluation of long-range electrostatics were used. C–H bonds were restrained using SHAKE [36]. A 2 fs timestep was used, and NPT production simulations were conducted at 300 K using a Langevin thermostat. Convergence was assessed by calculation of RMSD after removing global translation and rotation; in all cases this value had stabilized by the end of the unrestrained simulation portion of the equilibration protocol. Final production simulation lengths were as follows: TtAgo free protein (derived by removing the nucleic acid from the TtAgo 19 base pair ternary complex 3HK2), 100 ns; TtAgo 3HK2 fully-matched complex, 80 ns. TtAgo 3F73 12-bp mismatched ternary complexes G3T, A4C, A4T, G6C and T7G, 6 ns each.

The correlation analysis presented here is from Kong and Karplus [14], [15]. For each simulation trajectory, we computed the nonbonded pairwise residue interaction energies (electrostatic and van der Waals) for each snapshot. Since covalent bond energies do not strongly reflect the conformational state of the structure, only nonbonded energies were calculated. For each MD trajectory frame, the **residue interaction energy matrix** *E* was calculated as
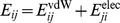
where |*i*−*j*|>1 to exclude interactions between adjacent (covalently bonded) residues. No periodic boundary condition or interaction distance cutoff was employed, and only solute residues were considered. The same force field parameters from above were also used here. As in the AMBER force field, no special treatment for dielectric screening effects was employed. Although this is an approximation [37], [38], the conclusions of this study are dependent primarily upon correlations among energies, not directly upon the energies themselves, and so we did not expect this to significantly affect the conclusions. We wrote software to perform this and subsequent steps.

Next, the subset of residue pairs whose average interaction energy was above a cutoff of 10 kcal/mol was selected. The 10 kcal/mol threshold was chosen for two reasons: 1) to eliminate low-level correlations, largely due to thermal noise, that are likely to have relatively little impact and 2) to limit the number of residue pairs for which correlations were calculated so that the computation could be done in reasonable time and memory. In the worst case, if all pairs are considered to have significant correlations, there are O(*n* ^2^) pairs. A correlation matrix is assembled from these pairs, which is therefore of size O(*n* ^4^). The threshold of 10 kcal/mol was selected so that on the order of 10^4^ pairs remained for each Argonaute structure. The correlation between each pair (*i*, *j*) and (*k*, *l*) of these pairs was then calculated:
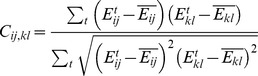
where the summations are over each frame *t* of the trajectory and overbars denote an average over the trajectory frames. Evaluating all selected pairs results in the **pair correlation matrix** C. Each row or column in the pair correlation matrix represented a pair of residues with average nonbonded interaction energy above the cutoff of 10 kcal/mol. Each pair correlation matrix cell represents the correlation over time of the respective nonbonded interaction energies of the two residue pairs. Most of these correlations were quite small. For example, in the fully-matched TtAgo 3HK2 structure, the 10 kcal/mol cutoff limited the size of the pair correlation matrix to 10392 pairs on each axis, compared to a worst-case size of roughly 33 billion on each axis. Even so, the average correlation value was 0.18 and only about 5% of pairs had an absolute correlation value greater than 0.5 (see Supporting Figure S1). The other structures we studied yielded similar statistics. Hence the number of significant correlations tended to be quite low.

This set of pair correlations is difficult to interpret as there is not a direct correspondence to structure. Hence, we project these correlations back onto the structure to create a **residue correlation matrix** *M*. This allows a concrete interpretation of the pair correlation matrix by mapping it directly onto structural elements. The correlation between residues *i* and *j* was defined as the sum of the correlations between each pair of two residue pairs *m* and *n* in *C*, but only if residue *i* was in *m* and *j* was in *n* (this constraint enforced with the delta function):
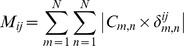
where *N* is the number of residues.

The **correlation factor** *F* between a residue *i* and some group of residues *G* was calculated as
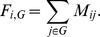
This allowed us to answer the question of how the sequence position of a nucleotide with residue number *i* is related to the degree of correlation it has to the part of the protein with residue numbers *G*. The correlation factor as a measurement was shown in the PDZ domain to be related to NMR chemical shifts resulting from binding of a ligand [39].

We calculated the **similarity** of two residue correlation matrices A and B as
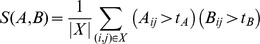
where *t*_A_ and *t*_B_ are the 92.6^th^ percentile value in *A* and *B* respectively (the rationale for this threshold is discussed in the Results section) and *X* is the set of indices (*i*, *j*) where either *A*_ij_ or *B*_ij_ are greater than their corresponding thresholds *t*_A_ or *t*_B_ respectively. This counts the number of corresponding cell pairs in *A* and *B* whose values are both above their thresholds. This count is divided by the number of cell pairs where at least one value is above its threshold. This has the effect of increasing the score for preserved large correlations, decreasing it for non-preserved large correlations, and ignoring unchanged small correlations. The maximum possible value of *S* is 1.

In order to determine the significance of a given similarity score, we wished to determine whether it was likely to be arrived at by chance. Two randomly selected Argonaute-like residue correlation matrices would have some expected similarity score. However, the distribution of *S* over all pairs of potential residue correlation matrices representing Argonaute proteins is not known. In order to estimate it, we used a bootstrap method employing randomized matrices to generate a distribution of scores. Each randomized matrix *A*′ was generated by randomly sampling rows and columns with replacement from an Argonaute residue correlation matrix *A*. This had the effect of randomly choosing a set of real correlations for each residue. Hence, the distribution mean is the expected similarity between two structures with randomly generated Argonaute-like residue correlations. For each combination of *A* and *B* for which we report a similarity score, we calculated *S*(*A*′, *B*′) one thousand times. The resulting distributions were roughly normally distributed and all were similar to each other. For TtAgo, each distribution had mean ∼0.1514 and standard deviation less than 0.001.

## Supporting Information

Figure S1 **Effect on residue correlation matrix of fully-matched nucleic acid binding to free TtAgo.** After each being normalized to the range [0,1), the free protein residue correlation matrix was subtracted from that of the fully-matched ternary complex (3F73). Note the organized pattern of change in residue correlation values. Only the protein is shown; residue indices are on each axis.
(TIFF)

Figure S2 **Residue correlation values, sorted by index, for each ternary complex.** Note two distinct regions, with transition at the same point for all ternary complexes.
(TIFF)

Figure S3 **Solvent-accessible surface area for each sensor residue in square Ångstroms.**
(TIFF)

Table S1 **Cancelling vdW vs electrostatic interactions for a representative snapshot of the fully-matched TtAgo ternary complex.**
(XLS)
