# Supplementary figures and images for: Convergent Transmission of RNAi Guide-Target Mismatch Information across Argonaute Internal Allosteric Network
Source: PLoS Comput Biol. 2012 Sep 27;8(9):e1002693. doi: 10.1371/journal.pcbi.1002693 (PMC3459882; doi:10.1371/journal.pcbi.1002693)

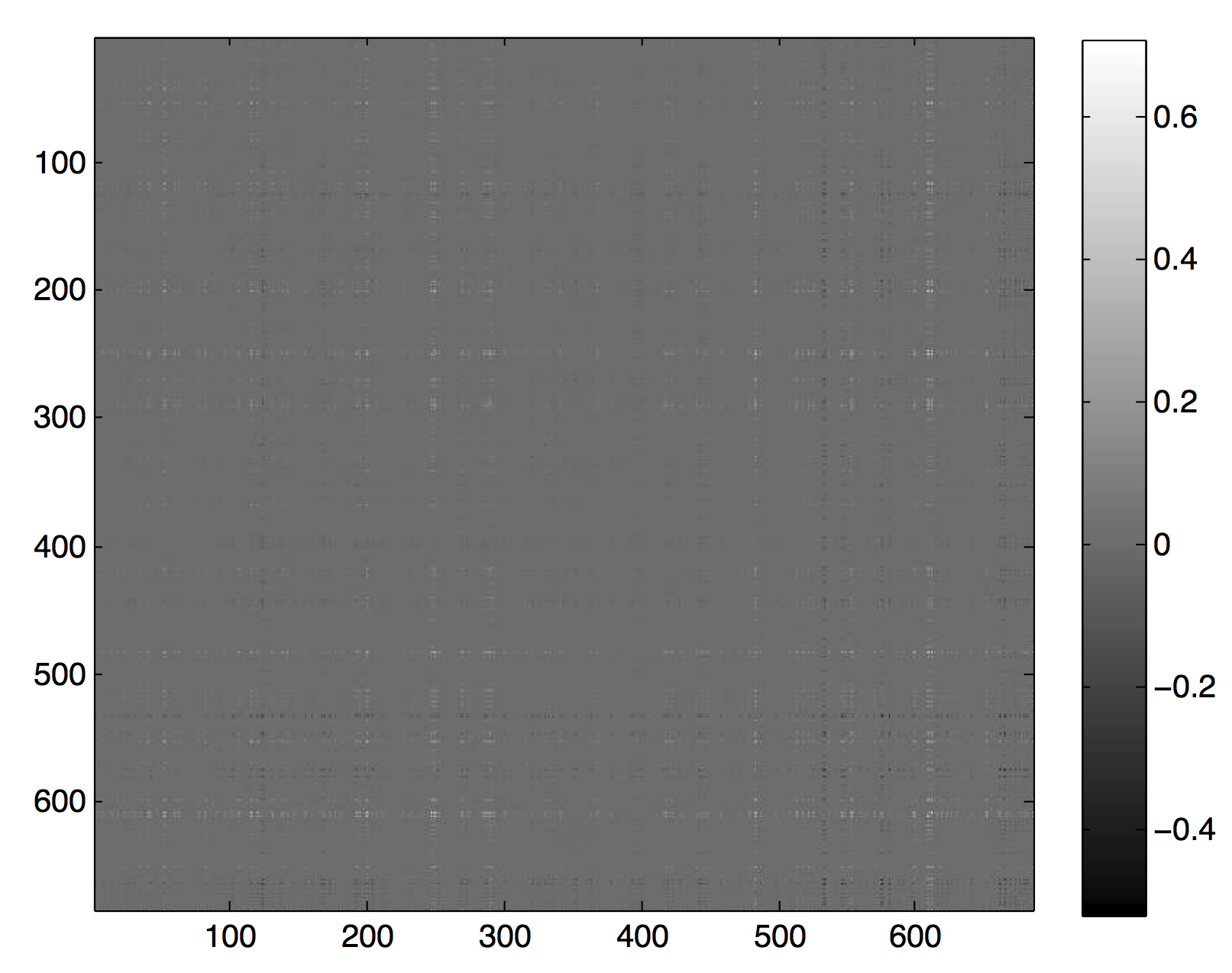

Supplement: Figure S1 — Effect on residue correlation matrix of fully-matched nucleic acid binding to free TtAgo. After each being normalized to the range [0,1), the free protein residue correlation matrix was subtracted from that of the fully-matched ternary complex (3F73). Note the organized pattern of change in residue correlation values. Only the protein is shown; residue indices are on each axis. (TIFF) [file pcbi.1002693.s001.tif]

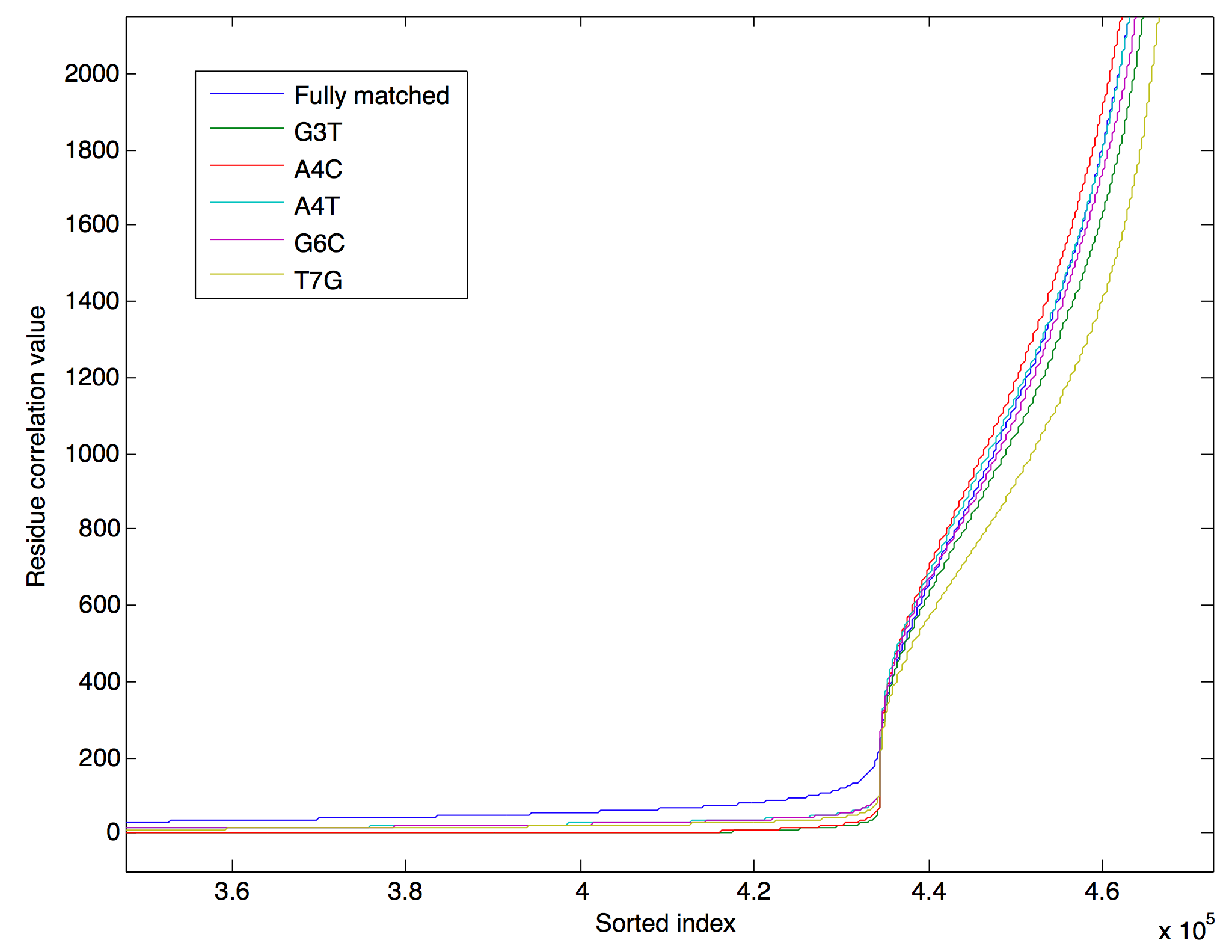

Supplement: Figure S2 — Residue correlation values, sorted by index, for each ternary complex. Note two distinct regions, with transition at the same point for all ternary complexes. (TIFF) [file pcbi.1002693.s002.tif]

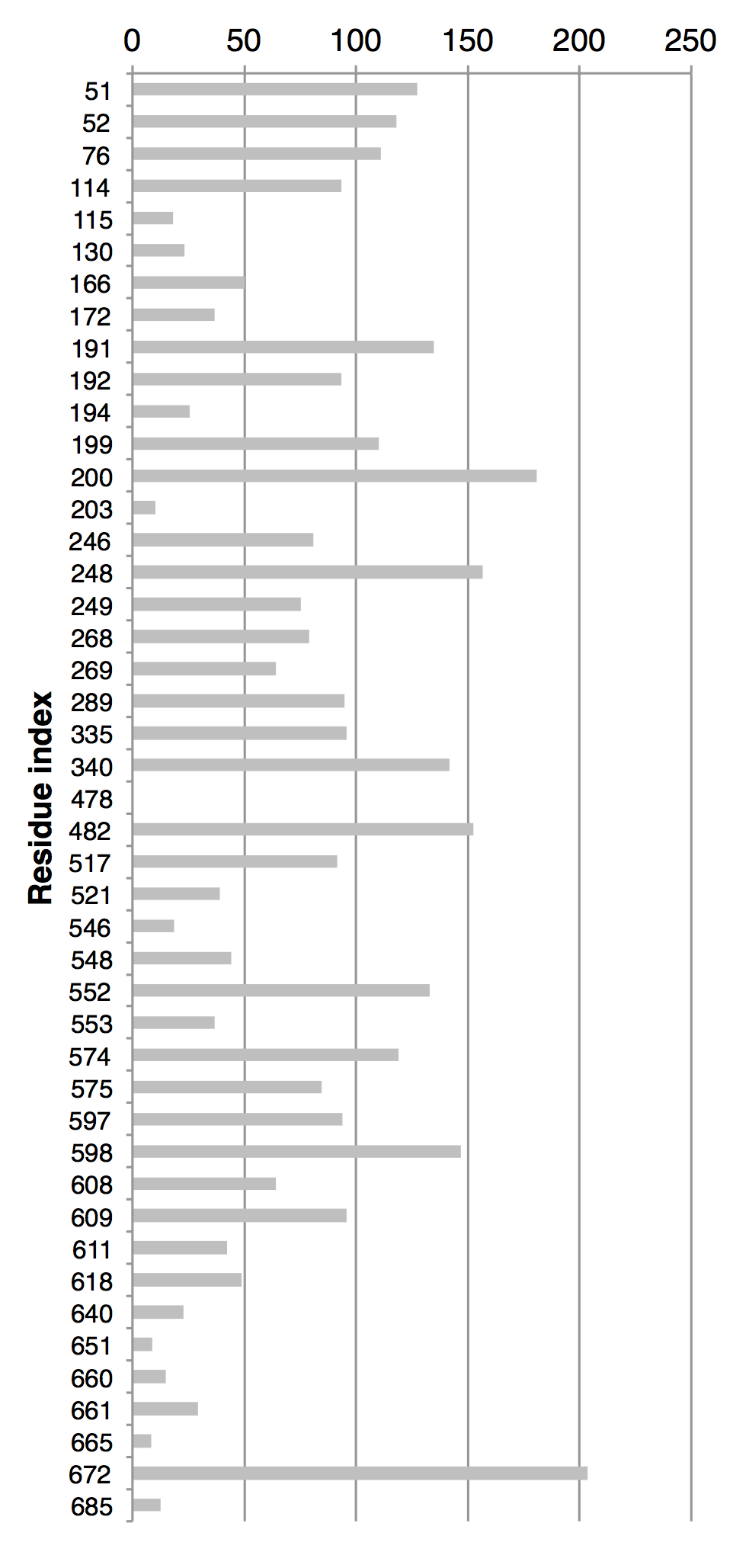

Supplement: Figure S3 — Solvent-accessible surface area for each sensor residue in square Ångstroms. (TIFF) [file pcbi.1002693.s003.tif]
